# Pneumatic Balloon Dilatation for Achalasia Cardia: Outcome, Complications, Success, and Long-term Follow-up

**DOI:** 10.5005/jp-journals-10018-1234

**Published:** 2017-09-29

**Authors:** Sudhir J Gupta, Nitin R Gaikwad, Amol R Samarth, Sonal R Gattewar

**Affiliations:** 1 Department of Gastroenterology, Government Medical College and Super Speciality Hospital, Nagpur, Maharashtra, India

**Keywords:** Achalasia, Eckardt score, Pneumatic balloon dilatation.

## Abstract

**Background:**

Achalasia is a chronic disease that can be managed with effective endoscopic modalities.

**Aim:**

To evaluate the effectiveness of single setting of pneumatic balloon dilatation for achalasia and assess the long-term success and outcomes.

**Materials and methods:**

This is a retrospective study of all achalasia patients who underwent pneumatic balloon dilatation at our institute. Patients who could be contacted were inquired regarding their symptoms and dysphagia-free interval after dilatation.

**Results:**

A total of 72 patients were enrolled. Out of this, 14 patients lost to follow-up. Mean age of 58 patients was 45.02 ± 16.51 years. Male:female ratio was 13:16. Mean predilatation Eckardt score was 7.16 ± 0.834. Type I achalasia was present in 10 (17.2%), type II in 44 (75.9%), type III in 4 (6.9%). Predilatation basal integrated relaxation pressure (IRP) was 28.14 ± 14.76 mm Hg. Postdilatation Eckardt score was 2.40 ± 1.67. Postdilatation dysphagia-free interval was 17.28 ± 6.70 months. A total of 9 patients (15.51%) failed to respond to first dilatation and 49 (84.48%) patients benefited from single setting of pneumatic dilatation. Esophageal perforation occurred in 2/58 (3.44%). We did not find any significant difference in gender distribution, age of presentation, duration of symptom, pre- and postdilatation Eckardt score, type of achalasia, and basal IRP on manometry between type of achalasia. Postdilatation dysphagia-free interval in type II achalasia (18.09 ± 5.976 months) was more than types I and III achalasia cardia (p = 0.066), which showed trend toward significance.

**Conclusion:**

Pneumatic balloon dilatation of achalasia cardia is a highly effective endoscopic procedure with minimal complications and mortality. Even the single setting of dilatation can have long-lasting dysphagia-free interval.

**How to cite this article:** Gupta SJ, Gaikwad NR, Samarth AR, Gattewar SR. Pneumatic Balloon Dilatation for Achalasia Cardia: Outcome, Complications, Success, and Long-term Follow-up. Euroasian J Hepato-Gastroenterol 2017;7(2):138-141.

## INTRODUCTION

Achalasia (a Greek word "failure to relax") is a rare disease with unknown etiology characterized by loss of esophageal peristalsis with failure of lower esophageal sphincter (LES) relaxation.^1^ Achalasia occurs equally in men and women with an incidence of 1 in 100,000 individuals annually and prevalence of 10 in 100,000.^2^ Symptoms of achalasia are intermittent dysphagia with/without weight loss. Various treatment options are pharmacological therapy (nitrates, calcium channel blockers), endoscopic pneumatic balloon dilatation, surgical management like Heller’s cardiomyotomy, and more recently peroral endoscopic myotomy (POEM). Each of these modalities has its own advantages and disadvantages in terms of success rate, ease of procedure, and complication rates. Among these, pneumatic balloon dilatation is highly efficacious and is commonly performed procedure in developing countries like India. We carried out this retrospective study to evaluate the efficacy and long-term follow-up after balloon dilatation in patients with achalasia cardia.

## MATERIALS AND METHODS

This was a retrospective study carried out at the Department of Gastroenterology, Government Medical College and Superspecialty Hospital, Nagpur, India. Institutional ethics committee approval was taken prior to the start of the study. Medical records of achalasia cardia patients who underwent pneumatic balloon dilatation at our institute from June 2013 to May 2016 were analyzed. All patients were contacted by outpatient department (OPD) visits/telephonically/personal interview. Patients were inquired regarding their symptoms and dysphagia-free interval after dilatation. All the demographic data were summarized from their previous records. Therapeutic success of pneumatic balloon dilatation was defined as Eckardt score of 3 or less.^3^

### Inclusion Criteria

**Figs 1A to C:**
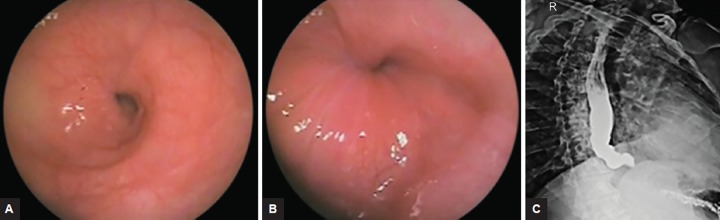
(A) Endoscopy showing dilated esophagus with liquid food residue; (B) tight gastroesophageal junction, not opening with air insufflation; and (C) barium swallow showing typical "birds beak appearance" of achalasia

**Figs 2A to C:**
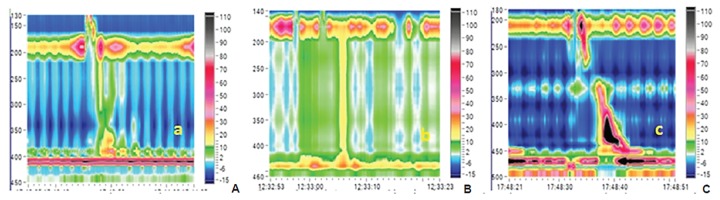
High resolution manometry: (A) Type I achalasia cardia; (B) type II achalasia cardia; and (C) type III achalasia cardia

Patients with proven achalasia cardia [clinically/endo-scopically/barium swallow/high-resolution manometry (HRM)] (Figs 1 and 2) who underwent pneumatic balloon dilatation in this Department from June 2012 to May 2016 were included in the study.

### Exclusion Criteria

Patients with a history of esophageal or gastric surgery and laparoscopic Heller’s myotomy were excluded.

### Statistical Analysis

The statistical analysis performed in the present study is descriptive and inferential. Continuous measurements are presented as mean ± standard deviation and categorical measurements are presented as number (%). The significance of study parameters on categorical scale between two or more groups was made by chi-square/ Fisher exact test. The significance of study parameters on continuous scale between two groups (intergroup analysis) on metric parameters was made by Student’s t-test. Data analysis was performed using the statistical software Statistical Analysis System version 9.2, Statistical Package for the Social Sciences version 15.0, Stata version 10.1, MedCalc version 9.0.1, Systat version 12.0, and R environment version 2.11.1.

## RESULTS

**Figs 3A and B:**
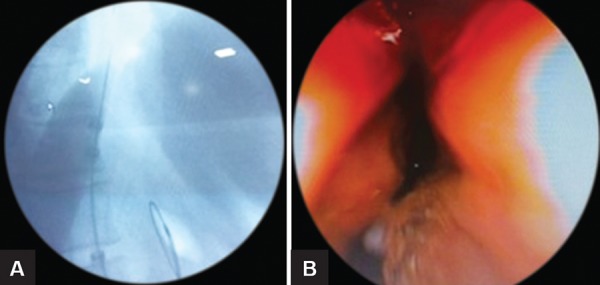
(A) Fluroscopic image of pneumatic ballon dilatation for achalasia cardia; (B) post dilatation endoscopic image.

A total of 72 patients were enrolled. Out of this, 14 patients were lost to follow-up and 58 patients were contacted by OPD visit/telephone/personal interview. Mean age of 58 patients was 45.02 ± 16.51 years. Total males were 26 and total females were 32. Male:female ratio was 13:16. Mean duration of symptoms was 12.47 ± 4.32 months. Dysphagia was present in 100% of patients with achalasia followed by regurgitation in 78.2%. Mean predilatation Eckardt score was 7.16 ± 0.834 (Table 1). Type I achalasia was present in 10 (17.2%), type II achalasia was present in 44 (75.9%), type III achalasia was present in 4 (6.9%) based on manometric data. Predilatation basal IRP was 28.14 ± 14.76. Postdilatation Eckardt score was 2.40 ± 1.67. Postdilatation dysphagia-free interval was 17.28 ± 6.70 months. Total 9 patients (15.51%) failed to respond to first dilatation and 49 (84.48%) patients benefited from pneumatic dilatation. Features and balloon dilatation have been shown in Figures 1 to 3. Complications in the form of esophageal perforation occurred in 2/58 (3.44%) patients, which were managed conservatively. Repeat dilatation was done in 3/58 (5.17%) patients who were symptomatic and were willing to undergo second setting of pneumatic dilatation. Successful dilatation was achieved in 6 (60%) in type I achalasia cardia, 40 (91%) in type II, and 3 (75%) in type III achalasia cardia patients. Different variables were compared in three types of achalasia but we did not find any significant difference in the form of gender distribution, age at presentation, duration of symptom, pre- and postdilatation Eckardt score, type of achalasia, and basal IRP on manometry. Postdilatation dysphagia-free interval in type II achalasia (18.09 ± 5.976 months) was more than types I and III achalasia cardia (p = 0.066), which showed trend toward significance.

**Table 1:** **Table 1:** Types of achalasia and study variables

		*Achalasia type I*		*Achalasia type II*		*Achalasia type III*		*p-value*	
Total patients		10 (17.2%)		44 (75.9%)		4 (6.9%)			
Age of patients in years		46.10 ± 13.683		44.14 ± 17.474		52.00 ± 12.754		0.651	
Male:Female		4:6		19:25		1:3		0.446	
Duration of symptoms in months		14.40 ± 4.300		12.02 ± 4.278		12.50 ± 4.655		0.296	
Eckardt score predilatation		7.00 ± 0.816		7.23 ± 0.859		6.75 ± 0.500		0.452	
Predilatation IRP basal mm Hg		23.790 ± 11.030		27.827 ± 14.674		42.575 ± 18.586		0.093	
Dysphagia-free interval postdilatation in months		15.90 ± 8.346		18.09 ± 5.976		11.75 ± 8.808		0.066	
Postdilatation Eckardt score		3.10 ± 1.792		2.20 ± 1.519		2.75 ± 2.872		0.0821	

## DISCUSSION

Achalasia is a rare primary esophageal motor disorder characterized by the absence of peristalsis and failure of relaxation of the LES. The peak incidence occurs between 30 and 60 years of age.^4^ The exact pathogenesis of achalasia is not known. It has been hypothesized that an autoimmune process triggered by a still unidentified cause results, in a genetically predisposed subject, in chronic inflammatory process leading to neuronal damage.^5^ In our study, most of the patients presented in the 4th and 5th decade and dysphagia was the most common symptom. In a study of Ghoshal et al,^6^ most common symptom of achalasia is dysphagia followed by regurgitation. The Eckardt symptom score is used for the evaluation of symptoms, stages, and efficacy of achalasia treatment; it includes symptoms like dysphagia, regurgitation, chest pain, weight loss.^7^ Mean Eckardt score in our patients was 7.16 ± 0.83, which reduced to the mean Eckardt score of 2.40 ± 1.67 after paneumatic ballon dilatation. The manometric findings of aperistalsis and incomplete LES relaxation without evidence of a mechanical obstruction indicate the diagnosis of achalasia.^8^ According to the Chicago classification, achalasia is divided into three subtypes. Type I: Absence of peristalsis, no pressurization within the esophageal body, high IRP. Type II: Absence of peristalsis, contractile activity, panesophageal pres-surization >30 mm Hg, and high IRP. Type III: Absence of peristalsis, and two or more spastic contractions with or without periods of compartmentalized pressurization. Type II achalasia is the most common type of achalasia worldwide. We also found type II being most common type of achalasia cardia in our study.^9^ Clinical presentation of all three types of achalasia is almost similar. In our study, type III achalasia patients are older than the rest two types, but this difference was not statistically significant. Roman et al^10^ showed the advanced age at presentation in type III achalasia. Average predilatation basal IRP in our patients was 28.148276 ± 14.76 mm Hg. Various treatment modalities involved in the management of achalasia are pharmacological treatment, endoscopic pneumatic balloon dilatation, Botulinum injection, Heller’s cardio-myotomy, and more recently POEM.^4^ Pharmacological treatment includes nitrates and calcium channel blockers, which decreases LES pressure to 60% and their effect is short lasting, hence, not much beneficial in achalasia.^11^ Pneumatic endoscopic dilatation (PD) uses air pressures to intraluminally dilate and disrupt the circular muscle fibers of the LES. Pneumatic endoscopic dilatation of the LES is considered the most effective nonsurgical treatment for achalasia. The most commonly used dilator is the microinvasive Rigiflex balloon system (Boston Scientific Corp, Boston, Massachusetts, USA). These balloons are available in three diameters (30, 35, and 40 mm). Accurate positioning of the Rigiflex balloon across the LES and obliteration of the balloon waist during fluoroscopy is important for effectiveness of the dilatation rather than the balloon distention time. The pressure required is usually 8 to 15 psi of air held for 15 to 60s. Studies suggest that by using the graded dilator approach, good to excellent relief of symptoms can be achieved in 50 to 93% of patients. Richter and Boeckxstaens^12^ showed relief of the symptoms in 74% by using 30 mm of balloon. In our study, the successful dilatation was achieved in 84.48% by using 30 mm balloon. In a European retrospective study in which serial dilatation was performed with the goal of reducing the LES pressure below 15 mm Hg, a 3-year success of 78 to 85% was reported with PD.^13^ The most serious complication associated with PD is esophageal perforation with an overall median rate in experienced hands of 1.9% (range 0-16%).^14^ In our study, esophageal perforation occurred in 2/58 (3.44%) patients, which were managed conservatively. Predictors of the best clinical outcomes after PD include age older than 40 years, women, LES pressure after dilatation 10 mm Hg, and type II pattern by HRM.^15^ Pneumatic endoscopic dilatation is the most cost-effective treatment for achalasia over a 5- to 10-year follow-up period.^16^ Laparoscopic Heller’s myotomy has almost same success rate as that of PD but with more morbidity.^17^ In our study, type II achalasia responded more to PD when the postdilatation dysphagia-free interval was compared with other two types. Treatment outcomes of achalasia subtypes according to manometry showed highest success for pneumatic dilatation in type II achalasia.^18^ The limitations of this study are as follows: First, treatment options, timing of treatment, and follow-up period in each patient were different because this is a retrospective study. The enrolled patients’ symptoms were recorded by the same planned description format. Second, this study has a limitation stemming from its small sample size.

In conclusion, pneumatic balloon dilatation of acha-lasia cardia is highly effective modality with minimal complications and mortality. Even the single session of dilatation is successful in maintaining long-lasting dysphagia-free interval. We conclude from our study that, though the era of POEM has already arrived, pneumatic balloon dilatation is still the most commonly performed endoscopic procedure for achalasia, especially in resource-poor developing countries.
